# Foraging Parameters Influencing the Detection and Interpretation of Area-Restricted Search Behaviour in Marine Predators: A Case Study with the Masked Booby

**DOI:** 10.1371/journal.pone.0063742

**Published:** 2013-05-22

**Authors:** Julia Sommerfeld, Akiko Kato, Yan Ropert-Coudert, Stefan Garthe, Mark A. Hindell

**Affiliations:** 1 Institute for Marine and Antarctic Studies, University of Tasmania, Hobart, Tasmania, Australia; 2 Research and Technology Centre, University of Kiel, Büsum, Germany; 3 Université de Strasbourg, IPHC, Strasbourg, France; 4 CNRS, UMR7178, Strasbourg, France; CNRS, Université de Bourgogne, France

## Abstract

Identification of Area-restricted search (ARS) behaviour is used to better understand foraging movements and strategies of marine predators. Track-based descriptive analyses are commonly used to detect ARS behaviour, but they may be biased by factors such as foraging trip duration or non-foraging behaviours (i.e. resting on the water). Using first-passage time analysis we tested if (I) daylight resting at the sea surface positions falsely increase the detection of ARS behaviour and (II) short foraging trips are less likely to include ARS behaviour in Masked Boobies *Sula dactylatra*. We further analysed whether ARS behaviour may be used as a proxy to identify important feeding areas. Depth-acceleration and GPS-loggers were simultaneously deployed on chick-rearing adults to obtain (1) location data every 4 minutes and (2) detailed foraging activity such as diving rates, time spent sitting on the water surface and in flight. In 82% of 50 foraging trips, birds adopted ARS behaviour. In 19.3% of 57 detected ARS zones, birds spent more than 70% of total ARS duration resting on the water, suggesting that these ARS zones were falsely detected. Based on generalized linear mixed models, the probability of detecting false ARS zones was 80%. False ARS zones mostly occurred during short trips in close proximity to the colony, with low or no diving activity. This demonstrates the need to account for resting on the water surface positions in marine animals when determining ARS behaviour based on foraging locations. Dive rates were positively correlated with trip duration and the probability of ARS behaviour increased with increasing number of dives, suggesting that the adoption of ARS behaviour in Masked Boobies is linked to enhanced foraging activity. We conclude that ARS behaviour may be used as a proxy to identify important feeding areas in this species.

## Introduction

Marine predators usually forage in an environment where food resources are patchily distributed in space and time [1]. Foraging success in marine predators depends largely on the distribution of their prey and the search methods employed to find them [2]. It is generally assumed that animals should spend more time in areas where resources are relatively plentiful [3]. Accordingly, individuals are expected to adjust their travelling behaviour in response to prey density and maximise their prey encounters by increasing turning rates and reducing travel speeds [4]. This adaptive response to prey density is generally referred to as Area-restricted search (ARS) behaviour [4].

Identifying ARS behaviour has been central to understanding how animals move through the environment in an effort to optimise their foraging success. In addition, understanding where and how animals allocate their time at sea is fundamental to predicting how animals may respond to changes in the marine environment [5], [6] and for management and conservation plans (e.g. [7], [8]). Several analytical methods have been developed in recent years to identify ARS behaviour in animals (e.g. [3], [9]–[11]). First-passage time (FPT) analysis, by definition the time an animal requires to cross a circle of a given radius along its trajectory [3], has been applied to identify ARS behaviour in studies investigating foraging movements in marine mammals, such as Elephant Seals *Mirounga leonina* (e.g. [12], [13]), as well as in numerous seabird studies across a wide range of species (e.g. [14]–[17]). Another track-based descriptive analysis is the fractal landscape method, which is similar to FPT analysis, but uses fractal dimensions instead of time [9], [10]. Finally, state-spaced models are also used to fit correlated random walks [18], [19] to animal tracking data [11], [20], [21]. In all these different analytical approaches, travel speeds and turning rates play a key role in detecting areas in which animals increase their search effort [22], [23].

Many marine predators spend a significant proportion of their foraging time resting at the sea surface (e.g. Northern Fur Seals *Callorhinus ursinus* [24]; Gray Seals *Halichoerus grypus* [25]; Baltic Ringed Seals *Phoca hispida botnica* [26]; Northern *Morus bassanus* [27] and Cape Gannets *M. capensis* [28]). These sea surface positions may, regardless of the analytical method employed, lead to inaccurate detections and interpretations of foraging movements in marine predators, including ARS behaviour. The reason behind this is that sea surface positions can rarely be properly quantified based solely on location. It is, for instance, very difficult if not impossible, to differentiate between plain roosting behaviour and potential in-water positions that may be associated with diving activity. Several studies addressed the influence of in-water positions on the detection of ARS behaviour by considering bouts on the water as flying bouts (e.g. [14], [17]). However, bouts on the water were estimated based on flight speeds between locations, potentially obscuring the duration of true resting phases. In other studies, only the nocturnal part of the foraging trip was removed (e.g. [16], [27]). This underlies the need to investigate foraging movements of marine predators in combination with time budgets. The detection of ARS behaviour can further be biased through foraging trip duration, because the shorter a foraging trip, the lower the number of locations available to identify ARS behaviour [15].

Studies comparing ARS behaviour based on location data with known diving events or prey captures are still scarce in foraging ecology (but see [12]–[14], [16]). In Wandering Albatross *Diomedea exulans*, no evidence was found that prey capture, measured by means of stomach-temperature loggers, was linked with ARS behaviour [14]. However, a strong link between the adoption of ARS behaviour and increased dive rates has been found in Northern Gannets [16], suggesting that, at least in some species, ARS behaviour is related to enhanced diving activity.

Masked Boobies *Sula dactylatra* are suitable marine predators to investigate this matter. Firstly, they forage mainly by plunge diving, which facilitates the detection of feeding events by means of pressure recording data devices. Secondly, Masked Boobies alternate short foraging trips, lasting only a few hours, with long foraging trips, lasting several hours to several days. This dichotomy makes it possible to address the influence of trip duration on the detection and accuracy of ARS behaviour. In addition, Masked Boobies have a relatively large body mass (range 1.7–2.9 kg; [29], this study). This allows simultaneous deployment of GPS and depth-acceleration loggers to investigate ARS behaviour in combination with diving events, so as to precisely determine how birds allocate their time at sea.

This study therefore aimed to establish if ARS regions identified from the tracks of Masked Boobies were indicative of foraging. In particular,

(I) we investigated time budgets of individuals within detected ARS zones, including diving activity and amount of time spent resting at the sea surface;

(II) we determined which factors influence the detection of ARS behaviour;

(III) we examined whether ARS behaviour can be used as a proxy to identify important feeding areas;

(IV) and finally, we developed a decision tree based on our findings to determine incorrectly-detected ARS zones resulting from high proportions of resting at the sea surface positions.

## Materials and Methods

### Ethics Statement

This research study was carried out under permission of Norfolk Islands National Park (Project Permit No 2009/0003/01). The animal use protocol for this study was reviewed and approved by the Animal Ethics Committee of the University of Tasmania (Project Permit No A10756), meeting the requirements of the Australian Code of Practice for the Care and Use of Animals in Scientific Purposes (1997).

### Study Site and Species

The study was carried out on Phillip Island (29°02'S, 167°57'E) in the south-west Pacific Ocean. Phillip Island (190 ha) is part of the Norfolk Island Group (Australia), located approximately 1670 km north-east of Sydney, Australia and 1070 km north-west of Auckland, New Zealand. The Norfolk Island Group lies within the “Norfolk Island Seamounts area”, a unique marine ecosystem surrounded by a high number of seamounts [30]. Fieldwork took place between 12 February 2010 and 03 March 2010, and between 27 October 2010 and 10 November 2010, corresponding to the late and early breeding seasons of Masked Boobies respectively.

Approximately 300 Masked Booby pairs breed on Phillip Island [31], [32]. Females lay two eggs, but generally only one chick is reared through obligate siblicide [33]. All study animals were rearing chicks between 3 and 11 weeks of age during February and March 2010 and between 2 and 7 weeks of age during October and November 2010.

### Data Devices and Deployment

Foraging movements of Masked Boobies were studied using GPS data loggers (46×32 mm, 20 g, Earth & Ocean Technologies, Kiel, Germany) recording time, latitude and longitude every 4 minutes. GPS loggers were attached on the top of three central tail feathers using waterproof adhesive TESA® tape (Beiersdorf AG, GmbH, Hamburg, Germany), minimising the damage to the feathers upon retrieval [34]. The activity patterns of Masked Boobies were studied using cylindrical, four channel data loggers (M190–D2GT, 12 bit resolution, 53×15 mm, 17 g, Little Leonardo, Tokyo, Japan), which simultaneously recorded depth (1 second), temperature (1 second) and acceleration (0.0625 seconds) along two axes. The relative accuracy for the depth sensor was 0.1 m. The loggers contain an acceleration sensor measuring both dynamic (vibration) and static accelerations (gravity). Depth-acceleration loggers were attached using TESA® tape underneath three central tail feathers (in addition to the GPS logger attached on the top) to measure acceleration along two axes: surging acceleration along the longitudinal body axis and heaving acceleration dorso-ventrally (for details, see [35], [28]). Total attachment weight, including tape, was 55 g (range 45–60 g, n = 24), corresponding to 2.2% of female mean body mass (2381 g, range 2120–2870 g, n = 9) and 2.7% of male mean body mass (2072 g, range 1685–2465 g, n = 15), which is below the generally accepted 3% body mass threshold ([36], [37]; but also see [38]).

Chick-rearing adults (either the female or the male of a pair) were captured with a noose-pole and weighed using a spring balance (Pesola®, maximum deviation ±0.3% of load). Adults were sexed by voice and by measurements: males have a distinctive higher pitched voice than females and are smaller than females within a pair [39]. Individuals were colour-marked on the breast with a green or blue sheep crayon for identification of the equipped individual in the field at first capture and, if non-banded, adults were banded with a metal leg band (Darvic®, Australian Birds and Bats Banding Scheme). Adults were released immediately onto their nests after attachment of the loggers. Loggers were left on for 1–4 days before being retrieved. Only one adult per pair and nest was studied to minimise disturbance and individuals were never studied twice. No nests were deserted during the entire duration of the study.

### First-passage Time Analysis

First-passage time analysis was conducted using R source code developed by D. Pinaud, CNRS, France [40], with each track interpolated at 0.1 km [15]. First-passage time was then calculated at each interpolated location along the track for radii ranging from 0.1 to 25 km, incrementing at 0.1 km. By plotting the log-transformed variance as a function of the radius size (the log-transformation is required to ensure the variance is independent of the magnitude), ARS scales were identified for each individual foraging track as the maximum peak in the log-transformed variance [3]. With the function *lavielle* of R package *adehabitatLT* [41], foraging trips containing interpolated locations and FPT values were split into homogenous bouts (see [42]). This function performs a non-parametric segmentation of a time series (here, the foraging track of the individual) using the penalised contrast method of Lavielle [41], [43], [44]. Next, we used the function *chooseseg* (*adehabitatLT*) to estimate the number of segments of each foraging track and the function *findpath* (*adehabitatLT*) to find the limits of the FPT segments (i.e. start and end of the segments). All segments of lower FPT values were then identified as ‘‘movement’’ and all higher segments were identified as ARS behaviour (see details in [41]).

### Overnight Foraging Trips

Five individuals performed overnight foraging trips. Based on accelerometer information, these birds spent most of the night sitting on the water surface for long hours without any significant activity (Masked Boobies only dive during daylight hours). Accordingly, the nocturnal portions of these trips were excluded because these resting positions may falsely increase the FPT variance [14]. Potential resting at the sea surface positions occurring during daylight hours were not removed because (1) the primary intention here is to show to what extent the occurrence of ARS behaviour is affected by the amount of time individuals spend sitting on the water surface, and (2) because the removal of daylight in-water positions is likely to obscure diving events. Each day of an overnight trip was therefore treated as a separate foraging trip for FPT analysis and calculation of ARS behaviour.

### Data Analysis

Acceleration and dive data were analysed using IGOR Pro 6.21 (Wavemetrics software, Portland USA). The component of the gravity acceleration along the surging axis was firstly separated from the high-frequency component resulting from wing beat activity, using a low-pass filter [45]. Then, the following types of behaviour were identified from the depth-acceleration logger signals: (1) on land, (2) take-off, (3) in flight (flapping/gliding), (4) sitting on the water surface and (5) plunge-diving. Diving threshold was set to 0.2 m. Flapping activity was identified as an oscillating pattern present simultaneously on both axes, with each propulsive stroke recorded on the heaving axis resulting in a forward acceleration recorded on the surging axis (see [28], [35]). The absence of these distinctive oscillation patterns within a flight bout corresponded to gliding activity. The acceleration on the surging and heaving axes together with the body angle adopted by the birds allowed us to differentiate between plunge-dives or dives from the surface. On-land sections were excluded from the analysis.

Each foraging trip was divided into individually numbered sections based on presence or absence of ARS behaviour. For example, if a foraging trip had one ARS zone present along the track, the trip was divided into three sections of known duration: (1) ARS absent, (2) ARS present and (3) ARS absent. If a foraging trip had two ARS zones, the foraging trip was divided into five sections of known duration: (1) ARS absent, (2) ARS present, (3) ARS absent, (4) ARS present and (5) ARS absent. For each section of a foraging trip, the proportions of time spent sitting on the water surface and in flight, as well as the total number of dives, were calculated.

Due to battery exhaustion of the depth-acceleration loggers, nine foraging trips were excluded from the data analysis and only complete foraging trips recorded by both GPS and depth-acceleration loggers are presented in the results section.

### Decision Tree for ARS Behaviour

We used a classification tree to construct a decision tree in R (package *tree* [46]) in order to identify ARS events in which birds spent a high proportion (>70%, see results) of the total ARS duration sitting on the water surface. Based on the analysis of the acceleration signals, such ARS events have a high probability of being falsely positive ARS zones. The decision tree was constructed to locate such *false* ARS zones in foraging trips of Masked Boobies for which only GPS information was available. ARS duration, scale of ARS zones (i.e. radius), mean distance of ARS zones from colony, as well as total foraging trip duration and total distance travelled (all parameters obtained through GPS devices) were used to classify *false* ARS. *Jack-knifing* [47] was used to estimate the performance of the decision tree. Briefly, *Jack-knifing* is a re-sampling method systematically re-computing (here, 100 times) the sampling set by leaving out one observation at a time, and using the resultant model to predict the nature of the excluded record.

### Statistical Analysis

All statistical analyses were performed using R 2.15.0 [40]. Generalized linear mixed models (GLMM [48]) were applied using the function *lmer* in R package *lme4* [49], with the occurrence or absence of ARS behaviour as a binomial response variable and the proportion of time birds spent sitting on the water surface (logit transformed), the number of dives, foraging trip duration and sex of the individual as fixed effects, with individual “trip ID nested within season” included as a random effect to account for pseudo-replication. The GLMM used a binomial error distribution with a logit-link function. Because the time adults spent sitting on the water surface was highly correlated with the time they spent in flight (z = –7.397, p<0.001, *τ* = –0.405, n = 155 *foraging trip sections*), the proportion of time adults spent in flight was excluded from the model. Prior to checking the fit of the model, model simplification and checking of model assumptions following [50], we tested whether a nested random effect “trip ID within season” improved the fit of the model by comparing the change in deviance of the model using *anova* (chi-squared under one degree of freedom), including the nested random effect to a second model with same fixed effects, but with only trip ID as a random effect. As p>0.05, we concluded that the nested random effect “trip ID within season” did not improve the fit of the model and used only individual trip ID as a random effect for further statistical testing. Bayesian inference from our simplified mixed model was obtained by applying the function *sim* of R package *arm* [51] to draw 1000 random values from the joint posterior distribution of the model parameters, taking the correlation between the parameters into account. Correlations were calculated using Kendall’s rank coefficient. To test for differences between foraging trip durations with and without ARS zones, a Welch’s t-test was performed. All tests were 2-tailed, with a significance level of p<0.05. Mean (±SD) results are reported.

## Results

### Foraging Trips

A total of 50 foraging trips from 26 individuals (10 females and 16 males) were recorded. Of the 26 individuals, 21 performed single-day foraging trips, while five adults (two females and three males) stayed overnight at sea, returning to the colony the next day. Masked Boobies had an averaged foraging trip duration of 6.52±3.79 h (range 0.88–14.18 h, n = 50) and travelled on average a total distance of 160.74±111.43 km (range 28.11–422.62 km, n = 50), at a mean maximum distance from colony of 74.41±68.06 km (range 10.68–250.39 km, n = 50) (Table 1). Trip duration was positively correlated with total distance travelled (z = 4.964, *τ* = 0.54, p<0.001, n = 50) and maximum distance from colony (z = 5.099, *τ* = 0.554, p<0.001, n = 50).

**Table 1:** Foraging parameters of Masked Boobies.

Foraging parameters		n
Trip duration (h)	6.52±3.79	50
	(0.88–14.18)	
Total distance travelled (km)	160.74±111.43	50
	(28.11–422.62)	
Maximum distance from colony (km)	74.41±68.06	50
	(10.68–250.39)	
Number of ARS per trip	1.1±0.8	50
	(0–4)	
Duration of ARS (h)	2.22±2.26	57
	(0.04–11.38)	
Scale of ARS (km)	3.51±3.95	57
	(0.3–16.9)	
Distance of ARS from colony (km)	63.89±62.77	57
	(9.18–244.5)	
% spent sitting on water within ARS	44.11±26.14	57
	(4.42–98.25)	
Number of dives within ARS	8.3±9.4	57
	(0–36)	

Values are mean ±SD. Ranges in parentheses. Sample size (n). Area-restricted search (ARS).

### Characteristics of ARS Zones

First-passage time analysis of the tracks revealed the presence of ARS behaviour in 41 out of the 50 foraging trips (82%), while in the remaining nine foraging trips, no ARS behaviour was detected. Foraging trips with and without ARS zones lasted on average 6.71±3.78 h (range 0.88–14.18 h, n = 41) and 5.67±3.93 h (range 2.06–13.30 h, n = 9), respectively. The duration of foraging trips with and without the presence of ARS behaviour were not significantly different (*Welch’s t-test* t = –0.726, df = 11.48, p = 0.482).

**Figure 1:**
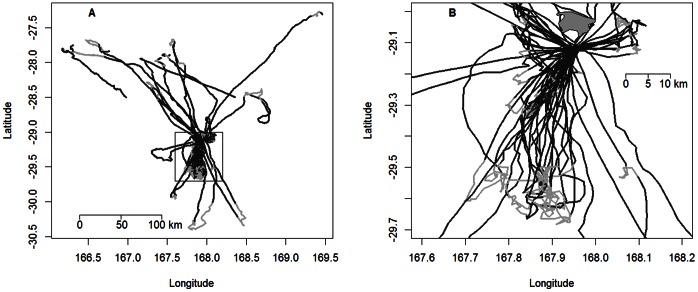
Foraging movements and Area-restricted search (ARS) behaviour of Masked Boobies recorded by GPS at 4 min intervals: (A) all foraging movements, and (B) foraging movements within close range of the colony. Light-grey lines correspond to ARS behaviour. Note: Foraging tracks ending or starting abruptly correspond to overnight foraging trips (see methods).

A total of 57 ARS events with an average of 1.1±0.8 ARS zones per foraging trip (range 0–4 ARS, n = 50) were detected in Masked Boobies (Fig. 1). Average ARS duration was 2.22±2.26 h (range 0.04–11.38 h, n = 57). The areas in which individuals increased their search effort were located at a mean distance of 63.89±62.77 km (range 9.18–244.5 km, n = 57) from the colony and had a mean scale (radius) of 3.51±3.95 km (range 0.3–16.9 km, n = 57) (Table 1). There was a significant positive correlation between mean distance of ARS zones from colony and scale of ARS (*Kendall* z = 2.943, p = 0.003, *τ* = 0.271, n = 57). Mean distance of ARS zones from the colony was further positively correlated with trip duration (z = 4.842, p<0.001, *τ* = 0.443, n = 57) and duration of ARS behaviour (z = 3.483, p<0.001, *τ* = 0.317, n = 57).

### Time Budget

A total of 537 dives were recorded, averaging 13.1±13.0 dives per foraging trip (range 1–48, n = 41). Of all dives, 65.2% (n = 350) occurred inside ARS zones, with a mean of 8.3±9.4 dives being observed per ARS zone (range 0–36, n = 57). Outside ARS zones, birds dived an average of 3.5±6.1 times (range 0–29, n = 98). In 10 out of 57 ARS zones (17.5%) no diving activity was recorded. All ARS events without dives were located less than 52 km from the colony, presented a smaller scale of ARS and were of shorter duration than ARS zones containing dives (Table 2). In addition, ARS behaviour with no diving activity was only observed during single day foraging trips.

**Table 2:** Foraging parameters of Area-restricted search (ARS) with respect to diving activity in Masked Boobies.

	ARS without dives(n = 10)	ARS with dives(n = 47)
Trip duration (h)	4.52±1.95	6.82±3.82
	(2.26–6.57, n = 7 trips)	(0.88–14.18, n = 39 trips)
ARS duration (h)	0.94±1.01	2.49±2.36
	(0.18–3.72)	(0.04–11.38)
ARS scale (km)	1.44±0.81	3.95±4.21
	(0.5–3.0)	(0.3–16.9)
Mean distance of ARSfrom colony (km)	18.45±14.19	73.56±64.91
	(9.18–51.63)	(10.23–244.52)
Sitting on watersurface (%)	63.19±24.08	40.01±24.95
	(31.11–94.31)	(4.42–98.25)

Values are mean ±SD. Ranges in parentheses. Sample size (n).

The time birds spent sitting on the water surface within ARS zones represented on average 44.11±26.14% of the total ARS duration (range 4.42–98.25%, n = 57), while outside ARS zones, birds spent a mean of 13.91±21.46% of their time sitting at the water surface (range 0.0–95.39%, n = 98). Inside ARS zones, flight time accounted for a mean of 52.68±25.63% of the total ARS duration (range 1.31–93.59%, n = 57), which was similar to the mean flight time outside ARS zones: 58.65±38.10% (range 1.29–100%, n = 98).

### Factors Influencing the Detection of ARS Behaviour

**Figure 2:**
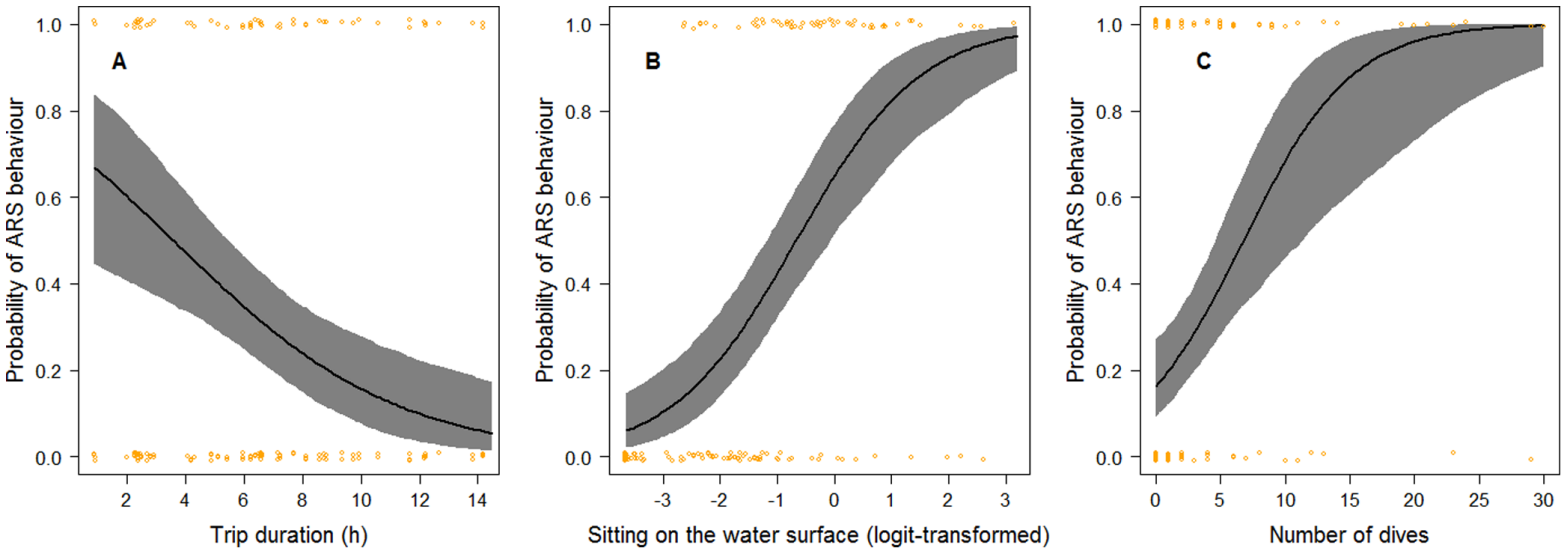
Predicted probability of Area-restricted search (ARS) behaviour in Masked Boobies in relation to: (A) foraging trip duration, (B) the logit-transformed proportion of time birds spent sitting on the water surface (a value of zero corresponds to 50%) and, (C) the number of dives. Bold lines represent the regression lines of the GLMM. Grey shaded areas represent the predicted 95% credible intervals. Orange circles are observed values.

The probability of ARS behaviour occurrence did not differ between sexes (GLMM, *b* = 0.478, z = 0.969, p = 0.332), subsequently the term *sex* was dropped from the model. The probability of detecting ARS behaviour increased significantly with increasing time that adults spent sitting at the water surface (GLMM, *b* = 1.214, z = 5.456, p<0.001) (Fig. 2) and with increasing number of dives (GLMM, *b* = 0.112, z = 2.487, p = 0.013) (Fig. 2). A significant interaction between the proportion of time adults spent sitting on the water surface and the number of dives indicated that the longer adults sat on the water surface and the less they dived, the more likely it was that FPT analysis detected ARS behaviour (GLMM, *b* = - 0.084, z = –2.427, p = 0.015). In contrast, a shorter time spent sitting on the water surface combined with a higher diving activity decreased the probability of detecting ARS behaviour. Surprisingly, the probability of an ARS event occurring decreased with increasing foraging trip duration (GLMM, *b* = − 0.261, z = –3.243, p = 0.001) (Fig. 2).

Based on our model, the predicted probability of detecting ARS behaviour in birds spending 50% of total ARS duration on the water surface was 0.65 (95% credible interval: 0.51–0.76) and 0.8 for birds spending at least 70% on the water surface (95% credible interval: 0.65–0.90) (Fig. 2). That is, the probability of ARS behaviour to be detected was greater than 0.8, if individuals sat for more than 70% of total ARS duration on the water surface, but less than 0.46 (95% credible interval: 0.35–0.57), if individuals sat for less than 30% of total ARS duration on the water surface (Fig. 2).

### *False* ARS Events and Decision Tree

By visual inspection of Figure 2 and based on the high ARS detection probability of 0.8, we defined ARS events in which birds spend more than 70% of the total ARS duration sitting on water as *falsely* positive ARS events. Therefore, 11 out of 57 ARS zones (19.3%) were regarded as *false* (Table 3).

**Table 3:** Foraging parameters of Masked Boobies with respect to the proportion spent sitting on the water surface within Area-restricted search (ARS) zones.

	Sitting on water 0–70% (n = 46 ARS)	Sitting on water 70–100% (n = 11 ARS)
Trip duration (h)	6.51±3.82	6.32±3.51
	(0.88–14.88, n = 36 trips)	(2.26–12.19, n = 9 trips)
Number of dives	7.09±7.96	2.18±4.19
	(1–30)	(0–14)
ARS duration (h)	2.20±2.23	2.28±2.49
	(0.04–11.38)	(0.39–6.85)
ARS scale (km)	3.97±4.24	1.58±1.27
	(0.3–16.9)	(0.4–4.0)
Mean distance of ARS from colony (km)	69.78±60.42	39.27±69.37
	(10.26–225.32)	(9.18–244.52)

Values are mean ±SD. Ranges in parentheses. Sample size (n).

*False* ARS events were typically located at short distance from the colony, of short duration and small size (Table 3). Diving activity of birds was overall low within these *false* ARS zones and in 5 out of 11 ARS zones birds did not dive at all (Table 4). Out of the remaining 6 ARS zones containing dives, only one female (trip ID 363.3.1) (Table 4) undertook an overnight foraging trip. First-passage time analysis detected a *false* ARS behaviour in this female at a mean distance of 244.5 km from colony, with ARS duration of 5.27 hours and a total of 14 dives within an ARS zone. The remaining *false* detected ARS zones occurred in single day foragers, within less than 56.6 km from the colony and a much lower diving rate (Table 4).

**Table 4:** Individual foraging parameters of Masked Boobies spending >70% of the Area-restricted search (ARS) duration sitting on the water surface.

Trip ID	TripDuration(h)	Max.distancefrom colony(km)	Totaldistancetravelled(km)	ARSduration(h)	MeandistanceARS fromcolony (km)	ARSsize(km)	Numberof dives	% sittingonwater	% inflight
18.2	2.92	11.0	36.49	0.68	9.18	0.6	0	92.3	7.7
70.2	8.74	15.71	68.53	6.85	14.69	4.0	1	76.4	21.9
70.3	6.57	36.64	113.84	1.41	15.34	0.8	1	71.3	26.3
70.3	6.57	36.64	113.84	1.07	10.28	0.8	0	71.4	26.3
272.1	9.73	65.92	168.74	1.63	56.62	0.4	1	98.3	1.3
275.1	4.87	16.29	121.82	0.46	14.46	0.5	0	94.3	4.6
285.1	7.17	19.38	44.16	6.09	18.81	1.4	5	95.2	3.5
287.1	2.39	16.58	37.62	0.82	11.53	1.4	0	72.3	26.5
287.1	2.39	16.58	37.62	0.40	11.45	1.4	0	83.6	14.3
290.2	2.26	37.77	77.76	0.45	25.13	2.3	2	81.3	17.5
363.3.1	12.19	250.39	275.19	5.27	244.52	3.8	14	80.5	17.7

Individual foraging trips (Trip ID). Note: Two distinct ARS zones were recorded in foraging trip “70.3” and “287.1”.

**Figure 3:**
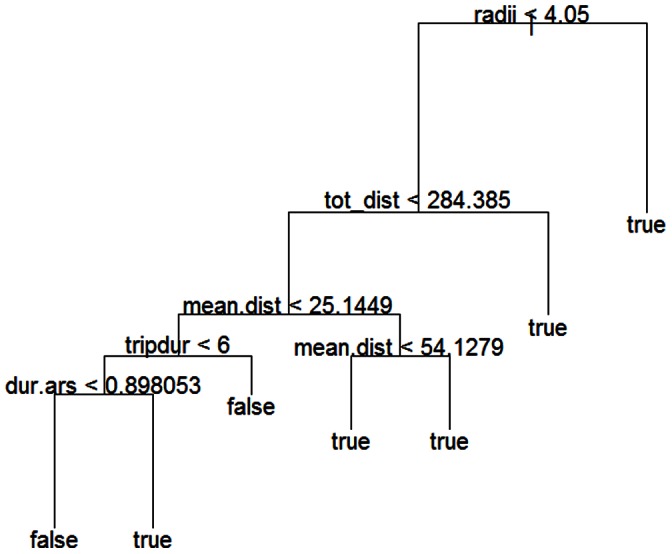
Classification tree for Area-restricted search (ARS) zones in Masked Boobies. ARS events in which birds spent more than 70% of total ARS duration sitting on the water surface are regarded as *false*. ARS events in which birds spent less than 70% on the water surface are regarded as *true*. Scale of ARS (radii), total distance travelled (tot_dist), mean distance of ARS from colony (mean.dist), foraging trip duration (tripdur) and duration of ARS behaviour (dur.ars). Distances and scale of ARS are given in km, duration in h.

Based on the decision tree, *false* ARS events were likely to be detected in ARS zones with a spatial scale <4.05 km, a total distance travelled <284.4 km, a mean distance of ARS from colony <25.14 km and a foraging trip duration >6.0 hours (Fig. 3). If foraging trips lasted less than 6.0 hours, *false* ARS zones were likely to be detected if ARS duration was less than 0.90 hours (Fig. 3). Through *jack-knifing* (see methods), 15 out of 21 *false* ARS (71.4%) and 76 out of 79 *true* ARS (96.2%) were estimated correctly.

## Discussion

This study aimed to establish which foraging parameters influence the detection of ARS behaviour in foraging locations of Masked Boobies and whether ARS zones were indicative of foraging. Our results provide strong evidence that resting at the sea surface positions leads to an incorrect detection and interpretation of approximately 19% of total ARS zones. This finding underlines the importance to account for resting at the sea surface positions when ARS behaviour is determined by means of foraging locations in marine predators and suggests that inferences should be drawn cautiously. In addition, we provide evidence that ARS behaviour is linked with enhanced diving activity, suggesting that ARS behaviour may be used as a proxy to identify important feeding areas in this species.

### Characteristics of ARS Zones

The adoption of ARS behaviour is expected to occur in patchy environments with high prey densities [4]. First-passage time analysis detected ARS behaviour in Masked Boobies in zones as close as 10 km from the colony, but also in pelagic areas as far as 240 km from Phillip Island. The scale and duration of ARS behaviour increased with increasing foraging trip duration. In addition, the longer the foraging trip, the more remote the location of ARS behaviour from the colony, suggesting that adults searched greater areas for prey over distant pelagic waters, than when foraging closer to the colony during short trips. This further suggests that prey densities within close proximity of the colony may have been lower (birds presented lower diving activity), than over distant pelagic waters (higher diving activity; see below). A significant positive relationship between the mean distance of ARS zones from the colony and the mean scale of ARS zones, as it was found in Masked Boobies, was also found across a range of long-distance foragers, such as Yellow-nosed Albatrosses *Thalassarche carteri* [52], Wandering Albatrosses [53] and Antarctic Petrels *Thalassoica antarctica* [54], but also in medium-ranging predators such as Northern Gannets [16].

### Time Budget and Factors Influencing the Detection of ARS Behaviour

The model containing the number of dives, the amount of time that individuals spent sitting on the water surface, as well as foraging trip duration, best explained the detection of ARS behaviour in Masked Boobies. Higher dive rates within ARS zones suggest that the adoption of ARS behaviour in Masked Boobies is linked with enhanced foraging activity. Consequently, ARS behaviour may be used as a proxy to identify important feeding areas in this species, in particular during foraging trips of longer durations (see below). Although prey capture rates were not estimated in Masked Boobies, it is reasonable to assume that at least some dives resulted in prey capture, as it was observed in Northern Gannets. In the latter, 43% of short and shallow V-shaped dives, in which birds used only the momentum of their fall to gain depth, and 95% of deep U-shaped dives, in which birds actively used their wings to reach greater depths and pursue prey, were associated with successful prey capture [55]. Masked Boobies exhibited higher dive rates inside ARS zones than outside ARS zones. The lower probability of birds adopting ARS behaviour when the number of dives was low, suggest that dives outside ARS zones are most likely opportunistic foraging events on isolated prey along their trajectory [14]. In addition, Masked Boobies may have quickly satisfied their energy requirements without adopting ARS behaviour, in particular during short foraging trips in close proximity to the colony [16]. A last possible explanation would be that the occurrence of dives outside ARS zones, are the result of unsuccessful dives. Foraging success in Southern Elephant Seals, inferred from *in situ* changes in relative body lipid content as derived from measured changes in buoyancy [56] was positively related to the probability of being in ARS mode, but high foraging success was also observed when individuals were in transit [12]. Outside these ARS zones, Elephant Seals moved quickly through areas without slowing down or increasing their turning rates [12]. This pattern has also been observed in Gray Seals [25], Basking Sharks *Cetorhinus maximus* [57] and Wandering Albatrosses when foraging in flight [14], suggesting that successful foraging without the adoption of ARS behaviour could be a common foraging method in marine predators.

Foraging trip duration influenced the accuracy of FPT analysis in other species [15]. The shorter a foraging trip, the lower the number of locations available for FPT analysis and the detection of ARS behaviour. In this study, we expected short trip duration to have a negative effect on the probability of ARS behaviour, however the opposite was actually observed. A possible explanation for this might be that the shorter the trip duration, the higher the proportion of time adults spent sitting on the water surface. As the probability of ARS behaviour increased significantly with increasing time spent sitting on the water surface, the probability of ARS behaviour might thus be higher in short foraging trips.

### *False* ARS Events and Decision Tree

First-passage time analysis cannot differentiate between areas in which individuals adopt slow travel speeds due to searching for prey or due to a resting pause at the sea surface. It is unlikely that Masked Boobies carried out surface-feeding events during these *false* ARS events, though such surface-feeding has been recorded in Cape and Northern Gannets [58], [59]. All dives recorded by data loggers in this study were plunge-dives and not dives initiated from the water surface. In addition, Masked Boobies have often been observed plunge-diving within less than 100 m of Phillip Island, but were never seen diving from the water surface. We suggest that *false* ARS events occurred mainly within close range of the colony because birds may have rested at the water surface after opportunistic or single diving events, which typically occurred without the adoption of ARS behaviour [16]. In fact, birds dived within an hour (range 1–4 dives) prior to *false* ARS events in 7 out of the 10 *false* ARS zones.

### Conclusions

Several analytical methods based on marine predators foraging movements have been developed (e.g. [3], [9]–[11]) to provide information on the location of foraging and feeding areas, which are otherwise difficult, if not impossible, to obtain through direct observation. Such comprehensive knowledge is ultimately needed to develop management and conservation plans for marine species and their habitats (e.g. [7], [8]).

We provide empirical evidence that the detection of ARS behaviour is highly influenced by the amount of time Masked Boobies spend sitting on the water surface during daylight hours, leading to an incorrect detection and interpretation of approximately 19% of total determined ARS zones. Without the information on individuals’ time budgets, we would have misleadingly assumed that the probability of ARS behaviour was higher during short foraging trips in the vicinity of the colony, than during long foraging trips at greater distances. This clearly demonstrates the need to account for time budgets during daily foraging movements in order to correctly identify ARS behaviour in marine predators. Nevertheless, although approximately 35% of the dives occurred outside ARS zones, the probability for ARS behaviour to occur was strongly linked with increased dive rates, which in turn were negatively correlated with the time spent resting on the water surface by adults. This suggests that ARS behaviour may be used as a proxy to identify important feeding areas in this aerial marine predator. This study was developed on foraging data of Masked Boobies, but the methods employed and our findings have implications for the detection and interpretation of ARS behaviour in other marine predators.
